# Fine Mapping and Characterization of an Aphid-Resistance Gene in the Soybean Landrace Fangzheng Moshidou

**DOI:** 10.3389/fpls.2022.899212

**Published:** 2022-06-15

**Authors:** Jing Yang, Guangyang Liu, Junyong Tang, Xiujun Wang, Yanling Diao, Yang Su, Dan Sun, Jiawei Shang, Yong Guo, Li-Juan Qiu

**Affiliations:** ^1^The National Key Facility for Crop Gene Resources and Genetic Improvement (NFCRI) and MOA Key Lab of Soybean Biology, Institute of Crop Science, Chinese Academy of Agricultural Sciences, Beijing, China; ^2^Institute of Crop Resources, Heilongjiang Academy of Agricultural Sciences, Harbin, China

**Keywords:** soybean aphid, resistance gene, fine mapping, NBS-LRR, gene expression

## Abstract

The soybean aphid poses a severe threat to soybean quality and yield by sucking phloem sap and transmitting plant viruses. An early-maturing and highly resistant soybean landrace, Fangzheng Moshidou, with markedly reduced aphid colonization has been identified by screening of aphid-resistant soybean accessions. In a population derived from the cross of Fangzheng Moshidou with the susceptible cultivar Beifeng 9, resistance was conferred by a single dominant gene. Three linked markers, Satt114, Satt334, and Sct_033, on chromosome 13 were identified by bulked-segregant analysis. Additional simple-sequence repeat and single-nucleotide polymorphism (SNP) markers were developed for gene mapping. The resistance of Fangzheng Moshidou was fine-mapped to the interval between the SNP markers YCSNP20 and YCSNP80, corresponding to 152.8 kb in the Williams 82 assembly 2 genome. This region was near the reported loci *Rag2* and *Rag5* but did not overlap the interval containing them. A unique haplotype is described for Fangzheng Moshidou that distinguishes it from soybean accessions PI 587972, PI 594879, and PI 567301B in the interval containing *Rag2* and *Rag5*. These results indicate that Fangzheng Moshidou harbors a novel gene at a tightly linked resistance locus, designated as *RagFMD*. Fourteen candidate genes were annotated in the fine-mapping region, including seven NBS-LRR genes, which are usually considered resistance genes in plant defense. Most of these candidate genes showed variations distinguishing the resistant and susceptible parents and some genes also showed differences in expression between the two parental lines and at several times after aphid infestation. Isolation of *RagFMD* would advance the study of molecular mechanisms of soybean aphid resistance and contribute to precise selection of resistant soybeans.

## Introduction

Soybean, *Glycine max* (L.) Merr, is a source of protein and oil worldwide. The yield of soybean is often threatened by soybean aphid (SA, *Aphis glycines* Matsumura). SAs colonize young leaves and tender stems of plants as adults and nymphs and suck phloem sap with piercing and sucking mouthparts. The chlorophyll of damaged soybean leaves disappears, forming bright yellow irregularly shaped macule that gradually expand and turn brown. Severely damaged plants show curly stems, yellow leaves, short plants, reduced branches and pods, and yield loss (Wu et al., 2004; Ragsdale et al., 2007; Beckendorf et al., 2008; Xiao et al., 2013). The whole plant may die if severely infected with aphids during seedling stage. Soybean aphids reduce soybean quality and can lead to yield losses in excess of 50% in China (Wang et al., 1994; Ragsdale et al., 2007). SAs also carry viruses, and transmit soybean mosaic virus and other plant viruses by secreting honeydew when feeding on soybean plants (Hill et al., 2001; Davis et al., 2005; Gildow et al., 2008; Tilmon et al., 2011; McCarville et al., 2014).

Control of SAs has relied mainly on insecticides (Tilmon et al., 2011). Long-term use of insecticides not only increases production costs, but damages the environment, increases insecticide resistance, and hastens aphid biotype differentiation (Hanson et al., 2017). Introduction of aphid-resistant soybean cultivars is a promising strategy for controlling SAs (Ragsdale et al., 2011; Hodgson et al., 2012). Since the invasion by SA of the United States in 2000, aphid-resistant soybean accessions including Jackson (PI 548657), Dowling (PI 548663) among others have been identified (Hill et al., 2004; Mensah et al., 2005; Mian et al., 2008b). With biological type differentiation of aphid, the resistance of some soybeans has gradually disappeared (Kim et al., 2008; Hill et al., 2010; Alt and Ryan-Mahmutagic, 2013), and new aphid-resistant soybeans are urgently needed. Additional resistant soybeans including K1639, Pioneer 85B97, and others have been reported (Diaz-Montano et al., 2006; Schapaugh et al., 2010; Bhusal et al., 2013, 2014). All of these soybeans can be valuable for soybean breeding programs as sources of aphid resistance.

Some aphid-resistance loci carried by aphid-resistant soybeans have been identified. The resistance genes *Rag* and *Rag1*, from respectively Jackson (PI 548657) and Dowling (PI 548663) were mapped to soybean chromosome 7 (Hill et al., 2004; Li et al., 2007; Kim et al., 2010a). *Rag2* from PI 243540 to PI 200538 (Kang et al., 2008; Mian et al., 2008a; Hill et al., 2009; Kim et al., 2010b) and *Rag5* from PI 567301B (Jun et al., 2012) were mapped to chromosome 13. PI 587972 and PI 594879 were also characterized as carrying one dominant gene located in the *Rag2* region (Fox et al., 2014; Hill et al., 2017). *Rag3*, a single dominant gene from PI 567543C, was mapped to chromosome 16 (Zhang et al., 2010). Two dominant aphid-resistance genes, *Rag6* and *Rag3c*, from wild soybean 85-32 were mapped to chromosomes 8 and 16 (Zhang et al., 2017). Recessive genes *rag1c* and *rag4* carried by PI 567541B, *rag1b* and *rag3* from PI 567598B have been identified (Mensah et al., 2008; Bales et al., 2013). Some quantitative-trait loci (QTLs) and single-nucleotide polymorphisms (SNPs) associated with SA resistance have been identified by QTL mapping and genome-wide association study methods (Jun et al., 2013; Hanson et al., 2018; Lamantia et al., 2019). Among them, *Rag1*, *Rag2*, *Rag3c*, and *Rag6* were fine-mapped to a very small interval, although none of them were isolated (Kim et al., 2010a,b; Zhang et al., 2017). These intervals all harbored nucleotide-binding site leucine-rich repeat (NBS-LRR) genes, which are considered resistance genes in plant defense.

Resistance to SA controlled by *Rag* genes was described to be overcome (Kim et al., 2008; Hill et al., 2010; Alt and Ryan-Mahmutagic, 2013); then, the use of new germplasm or combinations of different resistance genes in one genotype could ensure the durability of host resistance (Michel et al., 2011; Ajayi-Oyetunde et al., 2016). A soybean landrace, Fangzheng Moshidou, from northeast China showed high aphid resistance and early-maturing in our previous study (Liu et al., 2014). The objective of the present study was to characterize the inheritance of SA resistance in this accession, perform fine mapping, and identify candidate resistance genes.

## Materials and Methods

### Plant Materials

Fangzheng Moshidou (accession ZDD00326 in the National Crop Genebank of China) is a maturity group (MG) 0 soybean landrace collected from Heilongjiang province in China. Beifeng 9 is a MG 0 soybean cultivar that is susceptible to SA. A cross was made between Beifeng 9 and Fangzheng Moshidou to create genetic mapping populations. Eight F_1_ plants were grown in the field at Heilongjiang Academy of Agricultural Sciences (HAAS), Harbin, China. One hundred and eighty-two F_2_ seedlings were used for genetic analysis of aphid resistance in the growth chamber and for bulked-segregant analysis (BSA) (Michelmore et al., 1991). Another 130 F_2_ seeds were planted in the field for developing advanced generation mapping populations by pedigree selection. The F_5_ generation was grown in Harbin and 650 single plants were used for mapping resistance genes. Eight hundred and fifty-three F_5:6_ plants were derived for fine mapping from F_5_ plants that were heterozygous in the initially mapped region. Seeds from identified recombinants were harvested for phenotyping of their progenies in order to further confirm the phenotypes of these recombinants. PI 587972 and PI 594879 carrying *Rag2* and PI 567301B carrying *Rag5*, which were used for controls of published aphid resistance genes in haplotype analysis, were provided by the National Crop Genebank of China.

### Soybean Aphid Culture

Soybean aphids were collected from the field at HAAS. The SAs were maintained on a susceptible soybean cultivar Williams 82 in growth chamber at approximately 26/22^°^C day/night temperatures with 16 h light and 8 h dark daily.

### Aphid Resistance Assessment

Soybean aphid resistance assessment was performed in the growth chamber or a net house. Tests in the growth chamber were used mainly to evaluate the aphid resistance of 8 F_1_ plants, 182 F_2_ plants, and progenies of recombinants. Soybean plants were grown in 8 × 8 × 8-cm plastic pots with four plants per pot in a 3:1 mixture of fertile soil and perlite. Tests in the net house were used to evaluate aphid resistance in 650 F_5_ plants and 853 F_5:6_ plants. Plants were grown in rows with 10-cm spacing within rows and 50 cm between rows using an unreplicated, completely randomized design. Susceptible and resistant parental controls were planted every 10 rows and 30 plants per row. Soybean plants in the growth chamber and net house were each infested with two wingless aphids using a brush at the V1 growth stage (with one set of unfolded trifoliolate leaves) and the number of aphids per plant was recorded 3 weeks after infestation. Resistance evaluation followed Mensah et al. (2008) using a scale of 0–4 where 0 = no SAs; 0.5 = 0–10 SAs; 1.0 = 11–100 SAs; 1.5 = 101–150 SAs; 2.0 = 151–300 SAs; 2.5 = 301–500 SAs, 3.0 = 501–800 SAs; 3.5 = more than 800 SAs, plants growing slowly, and leaves curled slightly and yellow, without black mold; 4 = more than 800 SAs, plants growing slowly, and leaves severely curled and yellow with black mold. In qualitative analyses, plants with phenotypic ratings of 0–1 were assigned as resistant and plants with ratings of 1.5–4 as susceptible. For evaluating whether the resistance of Fangzheng Moshidou was dependent on initial aphid levels, the three initial densities of aphids (2, 10, and 50 aphids/plant) were introduced to incompletely unfolded leaves at the soybean V1 stage with three repeats for each treatment. The number of aphids per plant was recorded 3 weeks post-infestation and resistance rating for each treatment was evaluated.

### DNA Extraction and Polymerase Chain Reaction Amplification

Young leaves from each plant were collected in a 2-mL centrifuge tube with steel balls and rapidly frozen in liquid nitrogen. Genomic DNA was extracted by the modified CTAB method (Murray and Thompson, 1980) and a Nanodrop 1000 spectrophotometer (Thermo Fisher Scientific Inc., Waltham, MA, United States) was used to quantify DNA concentration. All DNA samples were diluted to 20 ng/μL with water for genotype identification. EasyTaq DNA Polymerase (Beijing Transgen Biotech, Beijing, China) was used for polymerase chain reaction (PCR) amplification according to the instructions.

### Bulked-Segregant Analysis and Linkage Mapping

A set of 580 SSR markers distributed over the soybean genome were used to screen polymorphic markers in the parental lines, and primer sequences of these SSR markers were retrieved from Soybase.^1^ BSA was performed on a resistant and a susceptible bulk formed by pooling equal amounts of DNA from 20 resistant to 20 susceptible F_2_ plants. The bulks and parental lines were genotyped with polymorphic SSR markers. Markers showing heterozygous genotypes in the resistant bulk and homozygous genotypes in the susceptible bulk identical to that of the susceptible parent were assigned as closely linked markers based on the principle of BSA.

Additional polymorphic SSR markers used for linkage mapping were developed based on the results of BSA and predicted positions of markers in the Williams 82 reference genome (see foot note text 2). Initial mapping was performed using F_2_ and F_5_ populations and the initial mapping interval was delimited by analysis of the genotypic and phenotypic data. SNP markers were also developed using whole-genome resequencing data of parental lines (accession SAMC286906 and SAMC287315 at the National Genomics Data Center^2^ lying in the initial mapping interval. Flanking sequences of these markers were retrieved from the database of *Glycine max* Wm82.a2.v1 genomic sequence in Soybase. Primers were designed on an online site^3^ and their sequences are listed in Supplementary Table 1. Genotyping was performed by PCR amplification, followed by polyacrylamide gel electrophoresis (for SSR markers) or sequencing (for SNP markers).

### Aphid Infestation and RNA Extraction

Plants of the parental lines were grown in a growth chamber (70–80% relative humidity, 25/22^°^C, 16/8 h light/dark) for infestation with SAs. Each seedling was infested with 15 aphids at the V2 growth stage (two sets of unfolded trifoliolate leaves). Leaves were collected before infestation (0 h) and in the early stage (8 and 24 h) and later stage (7 days or 168 h) following SA infestation. All SAs were brushed off leaves during sampling. Samples were immediately frozen in liquid nitrogen and stored at –80^°^C for RNA extraction. Total RNA was extracted from leaf samples of about 20 mg with Trizol reagent (Invitrogen, Carlsbad, CA, United States).

### Complementary DNA Synthesis and Quantitative Real-Time Polymerase Chain Reaction Analysis

First-strand complementary DNA (cDNA) was synthesized using 1 μg of total RNA with a Primer Script RT reagent Kit (Takara Bio Inc., Tokyo, Japan) according to the manufacturer’s instructions. Primers used for real-time PCR are listed in Supplementary Table 2. A total of 2 μl of the synthesized first-strand cDNA was amplified by PCR in 20-μl reaction mixtures using SYBR Select Master Mix on a ABI 7300 Real-time PCR system (Applied Biosystems, Thermo Fisher Scientific Corp., Waltham, MA, United States) with the following procedure: 95°C for 5 min, followed by 40 cycles of 95°C for 15 s, 60°C for 30 s, and 72^°^C for 31 s. The soybean *actin 11* gene was used as the reference gene. Melting-curve analysis was performed to ensure that the PCR products were unique. The values from three independent biological samples were averaged, and relative expression levels of selected genes were calculated by the 2^–ΔΔt^ method (Livak and Schmittgen, 2001). The expression levels of candidate genes in the fine-mapping region were compared between Fangzheng Moshidou and Beifeng 9, and during infestation of SA at four time points: 0 h (before aphid infestation) and 8, 24, and 168 h (7 days) after aphid infestation. Significance of differences between parental lines without aphid infestation and two time points in Fangzheng Moshidou or Beifeng 9 after SA infestation was evaluated by one-way analysis of variance.

### Haplotype Analysis

A genome-wide genotyping array containing 1,58,327 SNPs (Beijing Compass Biotechnology Co., Beijing, China) was used to examine the haplotypes of mapping regions in Fangzheng Moshidou, Beifeng 9, PI 587972 (*Rag2*), PI 594879 (*Rag2*), and PI 567301B (*Rag5*) using the Illumina platform (Illumina, San Diego, CA, United States). All genotype data was loaded into Genome Studio 2.0 (Illumina, San Diego, CA, United States) for raw-data normalization, clustering, and genotype calling. Flanking SNPs were collected based on the physical positions 30318939–31329840 on chromosome 13, and SNPs with minor allele frequency < 0.25, missing rate > 50%, and heterozygosity ratio > 50% were removed. Graphic representations of initial mapping region in all accessions were drawn with polymorphic SNPs using FlapJack (Milne et al., 2010).

## Results

### Evaluation of Fangzheng Moshidou for Aphid Resistance

**FIGURE 1:**
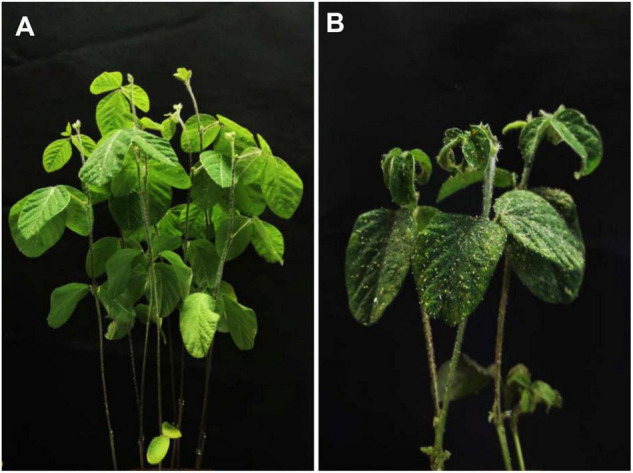
Phenotype of Fangzheng Moshidou and Beifeng 9 two weeks after SA infestation. **(A)** Resistant response of Fangzheng Moshidou seedlings infested with SAs; **(B)** Susceptible response of Beifeng 9 seedlings infested with SAs.

Fangzheng Moshidou showed high SA resistance in both growth chamber and net house. Two weeks after SA infestation, only a few aphids were observed on Fangzheng Moshidou plants, but many on Beifeng 9 plants (Figure 1). Fangzheng Moshidou also showed a resistance score of 1 in the net house test, whereas Beifeng 9 showed a score of 4. Furthermore, Fangzheng Moshidou showed high resistance after infestation even at initial density of 50 aphids per plant (Supplementary Table 3).

### Inheritance Pattern of Soybean Aphid Resistance in Fangzheng Moshidou

F_1_ and F_2_ plants of a cross between Beifeng 9 and Fangzheng Moshidou, together with the two parental lines, were tested for resistance to SA. The mean number of aphids on 12 plants of Fangzheng Moshidou was 21 after aphid infestation, whereas the mean number of aphids on 12 plants of Beifeng 9 was 358. The mean aphid number on eight F_1_ plants was 14 and all eight showed resistance to SA, suggesting that the resistance of Fangzheng Moshidou was controlled by a dominant gene or genes. Of the 182 F_2_ plants, 139 were resistant and 43 susceptible (Table 1). These frequencies fitted a 3:1 segregation (*x*^2^ = 0.117, *p* = 0.73), indicating that the aphid resistance of Fangzheng Moshidou was controlled by a single dominant *Rag* gene.

**TABLE 1:** Genetic analysis of aphid resistance in population of Beifeng 9 × Fangzheng Moshidou.

Parental lines and mapping populations	Observed numbers		Chi-square tests		
	Resistance	Susceptibility	Expected ratio	*x* ^2^	*p*
Beifeng 9	0	12			
Fangzheng Moshidou	12	0			
Beifeng 9 × Fangzheng Moshidou F_1_ plants	8	0			
Beifeng 9 × Fangzheng Moshidou F_2_ plants	139	43	3:1	0.117	0.73

### Initial Mapping by Bulked-Segregant Analysis and Linkage Analysis

**FIGURE 2:**
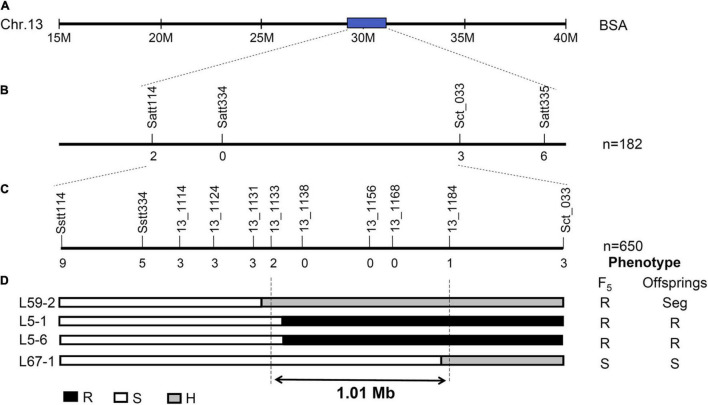
Initial mapping of SA resistance gene from Fangzheng Moshidou. **(A)** Chromosomal location of resistance gene identified by BSA. **(B,C)** Initial mapping of the resistance gene using F_2_ **(B)** and F_5_ **(C)** populations. Vertical lines indicate polymorphic SSR markers. Names of markers are shown above the line and the number of recombinants between gene and each marker is shown below the line. **(D)** The genotype and phenotype of recombinants used for restricting the mapping region. Black, white, and gray bars represent homozygous segments from Fangzheng Moshidou, homozygous segments from Beifeng 9, and heterozygous segments from the two parental lines, respectively. “Seg” of the phenotype represents segregation of resistance in the offsprings.

Of the 580 SSR markers distributed over the soybean genome, 180 (31%) were polymorphic between Beifeng 9 and Fangzheng Moshidou. These 180 markers were used to search for linked markers in the resistant and susceptible pools. Three markers (Satt114, Sat_033, and Satt335) located on chromosome 13 were identified as closely linked, all showing heterozygous genotypes in the resistant bulk and homozygous genotypes like those of the susceptible parent Beifeng 9 in the susceptible bulk. The polymorphic SSR markers in other regions were all heterozygous in both bulks. These three markers were then used to genotype all F_2_ plants and the putative gene conferring SA resistance was assigned to the interval between Satt114 and Sct_033. A set of SSR markers located in this mapping region were then tested for polymorphism between the two parental lines, yielding eight polymorphic markers: BARCSOYSSR_13_1114, 13_1124, 13_1131, 13_1133, 13_1138, 13_1156, 13_1168, and 13_1184. A set of 650 lines of the F_5_ population derived from the same cross were genotyped with these markers. These genotypic data together with the phenotypic data of the F_5_ plants and the progenies of recombinants localized the resistance gene between BARCSOYSSR_13_1133 and 13_1184 (Figure 2).

### Haplotype Analysis Indicated That the Resistance of Fangzheng Moshidou Was Conferred by a Novel Gene

**FIGURE 3:**
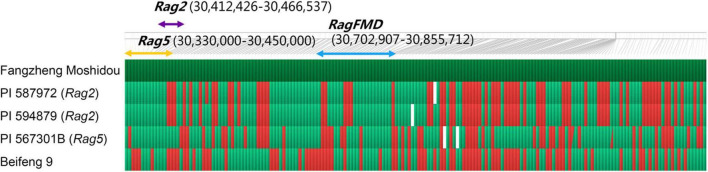
Haplotype analysis of the intial mapping region in different soybean genotypes. The SNPs of Fangzheng Moshidou, PI 587972 (*Rag2*), PI 594879 (*Rag2*), PI 567301B (*Rag5*), and Beifeng 9 were genotyped by a genome-wide genotyping array containing 158,327 SNPs and haplotypes of initial mapping region were carried out by FlapJack software. The reference line Fangzheng Moshidou is shown in dark green. Green, red, and white squares represent same, different, and missing genotypes from the reference, respectively. Purple, yellow, and blue arrows indicate mapping intervals of *Rag2*, *Rag5*, and *RagFMD*, respectively.

According to their physical locations, the dominant genes *Rag2* and *Rag5* on chromosome 13 were located in the primary interval identified in Fangzheng Moshidou, and the recessive gene *rag4* is far from this region. The haplotype of Fangzheng Moshidou proved distinct from those of the resistant lines PI 587972 (*Rag2*), PI 594879 (*Rag2*), and PI 567301B (*Rag5*). No other genotypes tested, including the susceptible Beifeng 9, carried this haplotype. PI 587972 and PI 594879 carrying the same resistance gene (*Rag2*) shared the same haplotype (Figure 3). The aphid-resistance gene in Fangzheng Moshidou was accordingly assigned as a new gene and designated as Resistance to *Aphids glycines* in Fangzheng Moshidou (*RagFMD*).

### Fine Mapping of *RagFMD* to a 152.8 kb Region

**FIGURE 4:**
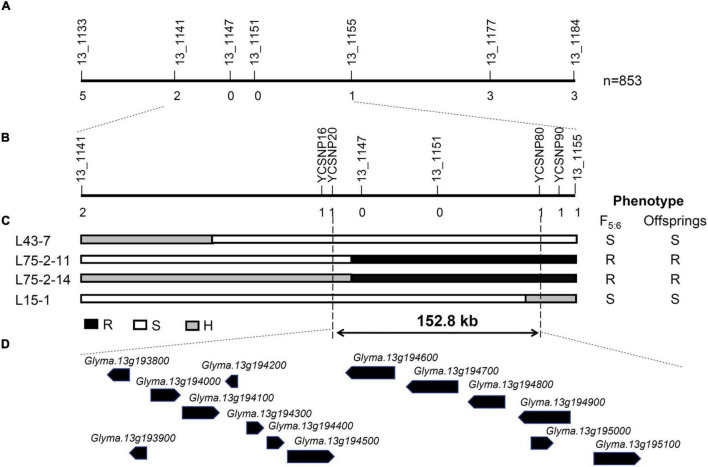
Fine mapping of *RagFMD* locus. **(A,B)** Fine mapping of *RagFMD* locus using F_5:6_ populations. Vertical lines indicate polymorphic markers. Names of markers are shown above the line and the number of recombinants between gene and each marker is shown below the line. **(C)** The genotype and phenotype of recombinants used for restricting the fine mapping region. Black, white, and gray bars represent homozygous segments from Fangzheng Moshidou, homozygous segments from Beifeng 9, and heterozygous segments from the parental lines, respectively. **(D)** Annotated gene models within 152.8 kb region based on the Williams 82 genome assembly (Wm82.a2.v1).

To further map the position of *RagFMD* locus, 853 F_5:6_ lines derived from F_5_ individuals heterozygous in the initially mapped region were genotyped using flanking markers BARCSOYSSR_13_1133, and 13_1184 and recombinants were identified. The additional SSR markers BARCSOYSSR_13_1141, 13_1147, 13_1151, 13_1155, and 13_1177 were used to genotype these recombinants and the mapping interval was narrowed to the region between markers BARCSOYSSR_13_1141 and 13_1155 (Figure 4). This region did not coincide with the mapping regions of other *Rag* genes including the nearby *Rag2* and *Rag5* (Kim et al., 2010b; Jun et al., 2012; Lee et al., 2017), further supporting its assignment as a new aphid-resistance locus. Four SNP markers (YCSNP16, YCSNP20, YCSNP80, and YCSNP90) between BARCSOYSSR_13_1141 and 13_1155 were then developed based on the resequencing data of the two parental lines. The combination of the genotypic and phenotypic data of recombinants and their progenies finally localized *RagFMD* to the region between markers YCSNP20 and YCSNP80, an interval of 152.8 kb (Figure 4).

### Candidate Gene Identification

Fourteen genes were annotated in the 152.8-kb interval (30,702,907–30,855,712 bp) of the *RagFMD* locus based on the *Glycine max* Wm82.a2.v1 genomic sequence (Table 2). Seven (*Glyma.13G194100*, *Glyma.13G194500*, *Glyma.13G194600*, *Glyma.13G194700*, *Glyma.13G194800*, *Glyma.13G194900*, and *Glyma.13G195100*) encoded resistance (R) genes with NBS-LRR domains. The other seven encoded other proteins including sigma factor binding protein 1, chaperone DnaJ-domain superfamily protein, ribosome recycling factor, mitochondrial inner membrane protease subunit 1 (IMP1), and albumin I chain b. Of these 14 genes, 13 harbored variations between the two parental lines according to the resequencing data and six harbored non-synonymous variations (Table 2). Eleven genes harbored variations and seven harbored non-synonymous variations between the SA-resistant parent Fangzheng Moshidou and the reference soybean Williams 82, an SA-susceptible soybean like Beifeng 9.

**TABLE 2:** Functional annotations and sequence variations of candidate genes in the interval of *RagFMD* locus.

Gene ID	Functional annotations	Variations between Fangzheng Moshidou and Beifeng 9		Variations between Fangzheng Moshidou and Williams 82	

		All SNPs	Non-syn SNPs	All SNPs	Non-syn SNPs
*Glyma.13G193800*	Sigma factor binding protein 1	13	0	13	0
*Glyma.13G193900*	Chaperone DnaJ-domain superfamily protein	29	6	9	2
*Glyma.13G194000*	Ribosome recycling factor	7	1	0	0
*Glyma.13G194100*	NB-ARC domain-containing disease resistance protein	12	1	1	1
*Glyma.13G194200*	Mitochondrial inner membrane protease subunit 1 (IMP1)	10	0	0	0
*Glyma.13G194300*	Albumin I chain b	9	0	1	0
*Glyma.13G194400*	Albumin I chain b	33	0	1	0
*Glyma.13G194500*	NB-ARC domain-containing disease resistance protein	41	4	1	0
*Glyma.13G194600*	Disease resistance protein (TIR-NBS-LRR class) family	7	1	61	12
*Glyma.13G194700*	Disease resistance protein (TIR-NBS-LRR class), putative	1	0	44	4
*Glyma.13G194800*	Disease resistance protein (TIR-NBS-LRR class) family	2	1	78	5
*Glyma.13G194900*	Disease resistance protein (TIR-NBS-LRR class) family	6	0	113	34 (one stop-gain)
*Glyma.13G195000*	NA	0	0	0	0
*Glyma.13G195100*	Disease resistance protein (TIR-NBS-LRR class), putative	5	0	19	1

**FIGURE 5:**
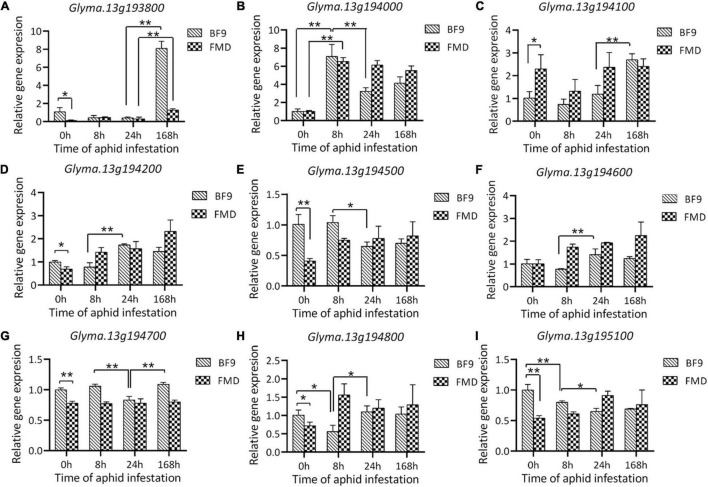
Relative expression analysis of the nine candidate genes **(A–I)** in Beifeng 9 and Fangzheng Moshidou after SA infestation. Fifteen aphids were introduced to soybean plants and the relative expressions of candidate genes were determined at 0, 8, 24, and 168 h (7 days) after infestation. FMD, Fangzheng Moshidou; BF9, Beifeng 9. Bar represents a standard deviation of three biological replicates. Significance of differences was evaluated by one-way analysis of variance. The * and ** represent significant difference (*P* < 0.05) and extremely significant difference (*P* < 0.01) between two labeled parts including either parental lines or time points in BF9 or FMD after SA infestation.

Expression of 5 genes (*Glyma.13g193900*, *Glyma.13g194300*, *Glyma.13g194400*, *Glyma.13g194900*, and *Glyma.13g195000*) was extremely low in both parental lines both before and after aphid infestation. Among the other 9 genes, four (*Glyma.13G193800, Glyma.13G194100*, *Glyma.13G194500* and *Glyma.13G195100*) showed significant expression differences between the two parental lines before aphid infestation (Figure 5). With aphid infestation, the expression levels of *Glyma.13G193800*, *Glyma.13G194000*, and *Glyma.13G194100* in Beifeng 9 were significantly changed. Among them, the expression of *Glyma.13G194000* was up-regulated at 8 h post-infestation and those of *Glyma.13G193800* and *Glyma.13G194100* was up-regulated at 7 days post-infestation. However, in Fangzheng Moshidou, the expressions of *Glyma.13G194000* and *Glyma.13G194800* were significantly up-regulated at 8 h post-infestation and that of *Glyma.13G193800* was up-regulated at 7 days post-infestation. In particular, *Glyma.13G193800* showed significant expression differences both before and after aphid infestation. The differentially expressed genes were assigned as candidate genes for further investigation.

## Discussion

In the present study, the aphid resistance of a Chinese soybean landrace, Fangzheng Moshidou, was characterized. A previous report (Diaz-Montano et al., 2006) suggested that the resistance of some soybean accessions is density-dependent, given that they were susceptible when infested with a high aphid density but resistant at lower densities. The SA resistance of Fangzheng Moshidou even at high aphid density suggests its application in soybean breeding. Because it shows early maturity (in MG 0), whereas most resistance sources reported previously (Fox et al., 2014; Hill et al., 2017; Natukunda et al., 2019) have longer growth durations, Fangzheng Moshidou is a promising resource for developing early-maturing SA-resistant soybean cultivars.

Soybean aphid resistance genes have been identified on chromosomes 7, 8, 13, and 16 of soybean (Hanson et al., 2018). Among them, *Rag2*, *Rag5*, and *rag4* were located on chromosome 13. The gene *rag4* is a recessive gene distantly linked to SSR markers BARCSOYSSR_13_0518 and 13_0572 (Mensah et al., 2008). *Rag2* from PI 243540 was first mapped on chromosome 13, and the aphid-resistance gene carried by PI 200538 was then mapped to the same physical position and proposed to be allelic to *Rag2*, which was fine-mapped to the 54-kb interval between 30,412,426 and 30,466,537 bp on chromosome 13 (Kang et al., 2008; Mian et al., 2008a; Hill et al., 2009; Kim et al., 2010b). *Rag5* was mapped to a 120-kb interval (Gm13:30.33–30.35 Mb) on chromosome 13, which coincides with the fine mapping region of *Rag2*, but they were from different soybean accessions and of different (antixenosis and antibiosis) resistance types (Jun et al., 2012; Lee et al., 2017). In the present study, *RagFMD* carried by Fangzheng Moshidou was also mapped to chromosome 13. Although the initial mapping region overlaps the interval containing *Rag2* and *Rag5*, the haplotype of this region in Fangzheng Moshidou differed sharply from those of the accessions carrying *Rag2* and *Rag5.* Moreover, *RagFMD* was fine-mapped to the 152.8 kb interval 30,702,907–30,855,712 on chromosome 13 in the Williams 82 reference genome sequence, a region differing from the fine-mapping region of *Rag2* and *Rag5*. For this reason, we propose that *RagFMD* is a novel aphid resistance gene and can be used to extend resistance durability by pyramiding with other resistance gene.

Nucleotide-binding site leucine-rich repeat proteins function in plant resistance to insects. Some NBS-LRR protein-encoding genes conferring resistance to aphids have been isolated. The *Mi-1* gene in tomato (Kaloshian et al., 1995; Vos et al., 1998) and the *Vat* gene in melon (Boissot et al., 2010, 2016; Dogimont et al., 2014) all have CC-NB-LRR structures and confer resistance to potato aphid (*Meloidogyne incognita*) and cotton aphid (*Aphis gossypii*), respectively. The planthopper, like SA, is an insect with piercing–sucking mouth parts, and can kill the rice plant and severely reduce rice yields. Several planthopper-resistance genes including *BPH9* (Zhao et al., 2016), *BPH14* (Du et al., 2009; Hu et al., 2017), *BPH18* (Ji et al., 2016), and *BPH26* (Tamura et al., 2014) have been isolated and shown to encode NBS-LRR-type proteins. CC and NB domains of BPH14 may act in rice planthopper resistance by activating the salicylic acid signaling pathway and defense-gene expression (Hu et al., 2017). In soybean, many NBS-LRR type genes have been proposed as candidate genes of *Rag* loci, although none have been isolated. Of 13 genes in the 115-kb interval of *Rag1*, one of two NBS-LRR genes was proposed as a candidate gene (Kim et al., 2010a). The only NBS-LRR gene (*Glyma13g26000*) among the 7 genes in the interval of *Rag2* was considered the most likely candidate gene (Kim et al., 2010b). *Rag3*, *Rag3b*, *Rag3c*, and *rag3* are all located at the same physical position and an NBS-LRR gene cluster was present in this interval (Zhang et al., 2017). For the candidate genes of *RagFMD* in our study, 7 genes belong to the NBS-LRR gene family and four showed non-synonymous variations between parental lines. The expression of three NBS-LRR genes was induced after aphid infestation in the aphid-resistant soybean Fangzheng Moshidou. These results further suggest that an NBS-LRR gene mediates aphid resistance in soybean.

Among the 14 annotated genes in the fine-mapped interval of *RagFMD*, 2 genes (*Glyma.13g194300* and *Glyma.13g194400*) encode albumin I chain b family proteins, which are orthologous to the toxic protein PA1b (Pea albumin 1, subunit b). PA1b is a small and compact 37-amino-acid protein isolated from pea (*Pisum sativum*) seeds. PA1b confers insecticidal activity against cereal weevils and aphid species, making it a promising bioinsecticide (Gressent et al., 2011; Rahioui et al., 2014). The survival rate of both cotton aphid and pea aphid decreased in response to feeding with the protein (Gressent et al., 2007). Whether these 2 soybean genes function in SA resistance awaits further study.

## Conclusion

A novel SA resistance gene was identified in the early-maturing soybean landrace Fangzheng Moshidou and fine-mapped to a 152.8 kb interval on chromosome 13. This region harbors seven NBS-LRR domain-containing proteins and two toxic-protein PA1b-like proteins. Most of these candidate genes showed variations between the resistant and susceptible parental lines and some were induced after aphid infestation. Isolating this gene by map-based cloning will shed light on the molecular mechanism of SA resistance and facilitate marker-assisted selection in soybean breeding programs.

## Data Availability Statement

The datasets presented in this study can be found in online repositories. The names of the repository/repositories and accession number(s) can be found in the article/Supplementary Material.

## Author Contributions

YG, GL, and L-JQ conceived and designed the experiments. JY, YG, GL, JT, XW, YD, YS, DS, and JS performed the experiments. YG, JY, and GL analyzed the data. YG, JY, GL, and L-JQ wrote and revised the manuscript. All authors read and approved the final version of the manuscript.

## Conflict of Interest

The authors declare that the research was conducted in the absence of any commercial or financial relationships that could be construed as a potential conflict of interest.

## Publisher’s Note

All claims expressed in this article are solely those of the authors and do not necessarily represent those of their affiliated organizations, or those of the publisher, the editors and the reviewers. Any product that may be evaluated in this article, or claim that may be made by its manufacturer, is not guaranteed or endorsed by the publisher.

## Footnotes

1
https://www.soybase.org/

2
https://ngdc.cncb.ac.cn/

3
http://primer3.ut.ee/
