# Supplementary material for: Fine Mapping and Characterization of an Aphid-Resistance Gene in the Soybean Landrace Fangzheng Moshidou
Source: Front Plant Sci. 2022 Jun 15;13:899212. doi: 10.3389/fpls.2022.899212 (PMC9240472; doi:10.3389/fpls.2022.899212)
Supplement: Supplementary file 1 [file Data_Sheet_1.doc]

**Supplemental Table 1.** Sequences of primers for SSR and SNP markers used in gene mapping

| **Molecular Markers** | **Forward primer sequence（5‘-3’）** | **Reverse primer sequence（5‘-3’）** |
| --- | --- | --- |
| Satt114 | GGGTTATCCTCCCCAATA | ATATGGGATGATAAGGTGAAA |
| Satt334 | GCGTTAAGAATGCATTTATGTTTAGTC | GCGAGTTTTTGGTTGGATTGAGTTG |
| Sct_033 | CTTTTAAATTATAATAGCATGATCT | TGCTAATTTAGATTACGTTATGT |
| Satt335 | CAAGCTCAAGCCTCACACAT | TGACCAGAGTCCAAAGTTCATC |
| BARCSOYSSR_13_1114 | GAGTTCGGCGAATAGGTTTT | CTTTCCCAGGTTGCAAAAAG |
| BARCSOYSSR_13_1124 | CCACGTTTGAGAGATCACACC | GCCTGTTAACACACTCATACTAAAAA |
| BARCSOYSSR_13_1131 | TCTCGCTGCAACACACAAGT | GCTGCATCAGAGAACAATGC |
| BARCSOYSSR_13_1133 | GCAATACACAAAACCATGCTAGA | GGATTTCGTTTAGGGAATCTTG |
| BARCSOYSSR_13_1138 | GATCCGATCCCCTCCTAAAT | CAACCACCCCAATTTTGTTC |
| BARCSOYSSR_13_1141 | ACGGTATGACATCGTCACCA | GAATGTTAACCTATGATCAACCAAGA |
| BARCSOYSSR_13_1147 | TCGGTTTCATTAACAATTCATTT | TCGAGTTTGAAATTGATCAGGA |
| BARCSOYSSR_13_1151 | GCCTTATAAACCTGATAGGCTG | TTACCCGGGATAGAAAAGCC |
| BARCSOYSSR_13_1155 | ATGGACCCTTCAGTTGCAGT | TGCTCGGTGTCATTTATTGC |
| BARCSOYSSR_13_1156 | TGTTCCGTACCAAAACGACA | ATTTCGCAATGACGAAGGAA |
| BARCSOYSSR_13_1168 | TATGGAGGCTGGCTTATGCT | CACATGTTTGACGCTGATGA |
| BARCSOYSSR_13_1177 | GGAGAACATTTGTCTCGAGGTT | CCTGAGAGGACTTGACTGGG |
| BARCSOYSSR_13_1184 | CCTCTGCCTCTCCACACTCT | GCAGGATGATCTGTGATTGTCT |
| YCSNP16 | CACAAGTGACAGTGTGACACAACA | GTTACAAACAGAGAGAATAAGCCAGT |
| YCSNP20 | GATTCCCCCTGCTGATGACA | GCCTCATCAACTCAACCCTTACT |
| YCSNP80 | TCAACACTTCAGCCTACTTTCCT | CATTTCTGGCTTCTCACCTGAT |
| YCSNP90 | CAGGGGGATGTCAGAAGTGTT | AATACAAAGGCTCAACTAAATATGATT |

**Supplemental Table 2.** Sequences of primers used for Real-time PCR

| **Gene Name** | **Forward primer sequence（5‘-3’）** | **Reverse primer sequence（5‘-3’）** |
| --- | --- | --- |
| *Glyma.13g193800* | GGGTGGATGTTGATGAGGGCA | AATCGTATTGAAGGTGTTG |
| *Glyma.13g193900* | CTGCCACCTCGACGTGGTGGCC | CTATTCTGGCGACAACAATC |
| *Glyma.13g194000* | ACTGCTTCGTTTCTCCTCTTAA | CACAAATTTCGCCTAACAAACG |
| *Glyma.13g194100* | GCCGCTCATTTATTCATGGAAT | TGCCAATGTCTCAATGCATATG |
| *Glyma.13g194200* | CACAATTTCTCTCATCCGGAAC | AGGACCAAGAATGATATGTGCA |
| *Glyma.13g194300* | CATGAAGAAAGGAAGTGGCAAT | AGTGCTTCAGAATCAGAGTCAA |
| *Glyma.13g194400* | CTTATGCTAGGCTTGCTCCTAT | CCTATAGGGACACAACGACAAT |
| *Glyma.13g194500* | TCACAAAACTAAGCTGCACTTC | TCAAATGCAACCTGAAGGAAAG |
| *Glyma.13g194600* | GGGTACTGGTAGAAGAACCTTC | TCGACGGAAAGATTGAAGGTTA |
| *Glyma.13g194700* | ATAAGAGCTTACTGATGCCCTC | CCCCTAATTTCTTGAAGATGCG |
| *Glyma.13g194800* | GTCACCAACAGATCAGGCTATA | TCTTTGCCTCTGAAGCTAAGAA |
| *Glyma.13g194900* | TGATCTTTGATGCTTTGAGCTG | TTGCCTTTTCTTGTCCAATGAG |
| *Glyma.13g195000* | AAACAGTCCTCAAACACACCTA | TGTGTTTAAGGACGATTGCTTG |
| *Glyma.13g195100* | AAGACCGAGTTTCAAAGGAGAT | ATCAACATCAACAGAGACCACT |

**Supplemental Table 3.** Resistances of different soybean genotypes after infestation with several levels of SAs

| **Materials** | **Resistance levels with different number aphids infestation** | | |
| --- | --- | --- | --- |
|  | **2 aphids/ plant** | **10 aphids/ plant** | **50 aphids /plant** |
| Fangzheng Moshidou | 1 | 1 | 1 |
| Beifeng 9 | 4 | 4 | 4 |
| Williams 82 | 4 | 4 | 4 |
